# How Can Community Data Be Leveraged to Advance Primary Health Care? A Scoping Review of Community-Based Health Information Systems

**DOI:** 10.9745/GHSP-D-23-00429

**Published:** 2024-04-29

**Authors:** Shivani Pandya, Lena Kan, Emily Parr, Claire Twose, Alain B. Labrique, Smisha Agarwal

**Affiliations:** a Center for Global Digital Health Innovation, Johns Hopkins Bloomberg School of Public Health, Baltimore, MD, USA.; b Department of International Health, Johns Hopkins Bloomberg School of Public Health, Baltimore, MD, USA.; c Welch Medical Library, Johns Hopkins School of Medicine, Baltimore, MD, USA.

## Abstract

**Background:**

Community-based health information systems (CBISs) can provide critical insights into how community health systems function, and digitized CBISs may improve the quality of community-level data and facilitate integration and use of CBISs within the broader health system. This scoping review aims to understand how CBISs have been implemented, integrated, and used to support community health outcomes in low- and middle-income countries (LMICs).

**Methods:**

Both peer-reviewed and gray literature were included; relevant articles were identified using key terms and controlled vocabulary related to community/primary health care, health information systems, digital health, and LMICs. A total of 11,611 total records were identified from 5 databases and the gray literature. After deduplication, 6,985 peer-reviewed/gray literature were screened, and 95 articles/reports were included, reporting on 105 CBIS implementations across 38 countries.

**Results:**

Findings show that 55% of CBISs included some level of digitization, with just 28% being fully digitized (for data collection and reporting). Data flow from the community level into the health system varied, with digitized CBISs more likely to reach national-level integration. National-level integration was primarily seen among vertical CBISs. Data quality challenges were present in both paper-based and digitized CBISs, exacerbated by fragmentation of the community health landscape with often parallel reporting systems. CBIS data use was constrained to mostly vertical and digitized (partially or fully) CBISs at national/subnational levels.

**Conclusion:**

Digitization can play a pivotal role in strengthening CBIS use, but findings demonstrate that CBISs are only as effective as the community health systems they are embedded within. Community-level data are often not being integrated into national/subnational health information systems, undermining the ability to understand what the community health needs are. Furthermore, stronger investments within community health systems need to be in place broadly to reduce fragmentation and provide stronger infrastructural and systemic support to the community health workforce.

## Abstract (precis)

Community-based health information systems (CBISs) play a critical role in generating and using community data to better understand community health needs; however, CBIS data are not effectively being integrated into routine national/subnational health information systems, limiting the scope of its use.

## Teaser

**Key Findings:**

- Community-based health information systems (CBISs) can provide an understanding of the health needs at the community level, especially for populations that have more constrained access to health care.
- Digitization can strengthen CBIS data collection, reporting, quality, and use compared to paper-based CBIS; however, challenges with transitioning from paper to digital systems, data entry errors, limited connectivity, lack of interoperability, poor standardization of data indicators, and inadequate training of the community health workforce are all factors that may impede its effectiveness.
- This review found that community-level health data are not being integrated into routine national or subnational health information systems and national/subnational-level integration was largely facilitated by being a vertically-focused/disease-specific CBIS (i.e., for malaria or HIV) and/or being digitized, limiting the ability to leverage community-level data to strengthen community health outcomes and achieve universal health coverage.

**Key Implication:**

- Strengthening CBISs requires strengthening community health systems. There needs to be alignment both in terms of country-led integration of community health services as well as how the digital environment can enable the collection of community-level data and its integration within the broader health system.

## BACKGROUND

Health for all, the notion that health is a human right and that primary health care (PHC) is key to the attainment of “Health for All,” was established as part of the Alma Ata Declaration of 1978.^1^ Since then, these concepts have been continually refined and reimagined in attempts to achieve them, which has been demonstrated and reaffirmed by the creation and global commitment to the Millennium Development Goals and the Sustainable Development Goals.^2^^,^^3^ Community health worker (CHW) programs improve access to and delivery of PHC services often in hard-to-reach and rural communities globally.^4^ For over 4 decades, evidence from low- and middle-income countries (LMICs) has demonstrated that such CHW programs have contributed to substantial improvements in health outcomes due to improvements in the delivery of quality PHC.^5^^–^^7^ To facilitate recording and reporting of services provided at the community level, often by CHWs, community-based health information systems (CBISs) have been developed. As defined by MEASURE Evaluation, CBIS refers to a system that “involves data collection, management, and analysis of health services that exist within a community outside of health facilities”; this system can comprise a combination of paper, software, hardware, processes, and people.^8^ Having timely access to data on community health is critical to understanding the needs of the community as well as the effectiveness of CHW programs. Despite CHWs historically gathering community-level data in paper-based registries, the use of these data is fairly limited. In most settings, CBISs remain weak, lacking adequate data quality and reporting to inform decision-making, and are often overlooked in efforts to strengthen community health systems and advance universal health coverage.^7^

The terminology and acronyms used for CBISs vary across settings. However, common examples of CBISs include community monitoring systems for maternal, newborn, and child health (MNCH) and PHC services, commodities and stock management (e.g., medication volumes and supplies) at the community level, disease-specific surveillance (e.g., malaria), or collection of community data for civil registration and vital statistics (CRVS) systems.^9^^,^^10^ CBISs differ from traditional health information systems (HISs) in that the primary data collection is performed at the community level, typically as part of a health service encounter, and reported to health facilities in an aggregate format. CBISs can be used to identify populations in need, address challenges with access and accountability, and improve equity. However, given that community health systems, in most contexts, are not sufficiently supported or invested in, the quality and use of CBISs and, subsequently, the improvement of community health programs has been impeded.^11^ Though CBISs have existed for several decades, there are few examples of CBISs that are well functioning, have good data quality, and are implemented at scale. A well-functioning CBIS that is nationally scaled and integrated would facilitate improvements in data quality, facilitate linkages across the health continuum, yield data to support evidence-based decision-making to improve health programming, and contribute to achieving universal health coverage. In recent years, the interest in digitizing the community health workforce has also provided new opportunities and approaches toward real-time capturing and reporting of community-level data for active decision-making. CBISs are being digitized in many countries and, when well designed, can increase the availability and use of community-level data in a timely and reliable way.^12^

A well-functioning CBIS that is nationally scaled and integrated would facilitate improvements in data quality, facilitate linkages across the health continuum, and yield data to support evidence-based decision-making to improve health programming.

Currently, there are limited data and evidence around the use of CBISs and how they have been implemented globally, especially within LMIC settings. The available literature has primarily focused on HIS broadly and does not consider the role of and challenges related to the inclusion of community-level data and services. Furthermore, there is a paucity of evaluations on CBISs—including the use of digitally optimized CBISs—in improving community-level data quality and use. The lack of evidence on the use of CBIS hinders the potential of its use, given the critical importance community-level data can provide toward improving PHC. To better understand how CBISs have been implemented in LMIC contexts, a scoping review was conducted to: (1) describe the existing CBIS implementations in LMICs; (2) document how data were collected and reported in paper-based, digital, and mixed CBISs; and (3) detail experiences and challenges with data quality and data use, with a specific focus on the role of digitization.

## METHODS

### Study Design

A scoping review was conducted to understand how CBISs have been implemented and used in LMICs. The review was conducted in accordance with the Joanna Briggs Institute Manual for Evidence Synthesis.^13^ The research team (LK, SP) worked closely with an information professional (CT) from the Welch Libraries at the Johns Hopkins University to conduct the scoping review.

### Search Strategy for Peer-Reviewed Literature

Search strategies were constructed to identify articles addressing the use of CBISs in LMICs. Databases selected for inclusion were PubMed, Embase, Scopus, the Cochrane Library, and the World Health Organization Regional Indexes. Searches were conducted on November 15, 2021. The initial search strategy was developed for the PubMed database by an information professional (CT), with input from the research team (Supplement 1). When the search was deemed sufficiently comprehensive, this search strategy was translated for the other 4 databases by CT. A combination of keywords and medical subject heading terms were used to support the retrieval of relevant results. Search terms included community, health information systems, health monitor, surveillance, and health management, among others.

Search results from all the databases were downloaded into EndNote,^14^ a citation manager, duplicate citations were identified and isolated, and the resulting unique set of citations was uploaded into Covidence,^15^ a systematic review manager, for title and abstract as well as full-text screening.

### Search Strategy for Gray Literature

Gray literature was identified through reviewing relevant repositories, including WorldWideScience.org, the International Initiative for Impact Evaluation, and implementing organization’s reports (i.e., BRAC, Living Goods, Digital Square, U.S. President’s Malaria Initiative, among others).

### Inclusion and Exclusion Criteria

For peer-reviewed articles to be included, they had to be original research articles, report on a CBIS, and be LMIC-based. LMIC categorizations were developed by the Johns Hopkins Welch Libraries and included the listing of all countries that are categorized as either low- and/or middle-income countries. The articles had to be in English, which limited the coverage of countries ultimately included, and be available as full text. Articles were excluded if the systems they reported included no community element and were solely health facility based. Articles reporting on electronic health records or electronic medical records were excluded.

For gray literature to be included, it had to report on a CBIS. Similar to peer-reviewed articles, if the information system being reported on had no community element, it was excluded.

### Screening of Articles

#### Title and Abstract Screening

For the peer-reviewed literature, CT uploaded articles from the search onto Covidence. Two researchers (LK and SP) conducted the title and abstract screening. The first 500 articles were screened together to support inter-rater agreement; the remaining articles were independently screened. Disagreements were reviewed and addressed by a third reviewer (SA).

#### Full-Text Review

Included articles from the title and abstract screening were migrated to an Excel spreadsheet for the full-text review. LK and SP independently conducted the full-text review. Articles where the reviewers were unsure were discussed between them; if a decision could not be reached, SA was consulted to provide the final decision.

### Data Abstraction

Upon completing the full-text review, the included peer-reviewed articles and the gray literature were combined. A data charting form was created in Microsoft Excel, and data were abstracted from the included articles (peer-reviewed and gray literature) onto the form. The use of a data charting form is recommended as a key component of scoping reviews because it is an iterative process that facilitates a robust understanding of the data.^13^ Data abstracted from each study included contextual information about the study (authors, year of publication, type of article, and type of study), contextual information about the CBIS (country or countries of implementation, organization(s) involved in the implementation, type of CBIS; purpose of CBIS, and health domain); process of CBIS use (how data were collected, reported, and used in the CBIS), and enablers and challenges reported around use of CBIS. Three authors (SP, LK, EP) abstracted and reviewed the information on the data charting spreadsheet.

## RESULTS

### Search Results

**FIGURE 1:**
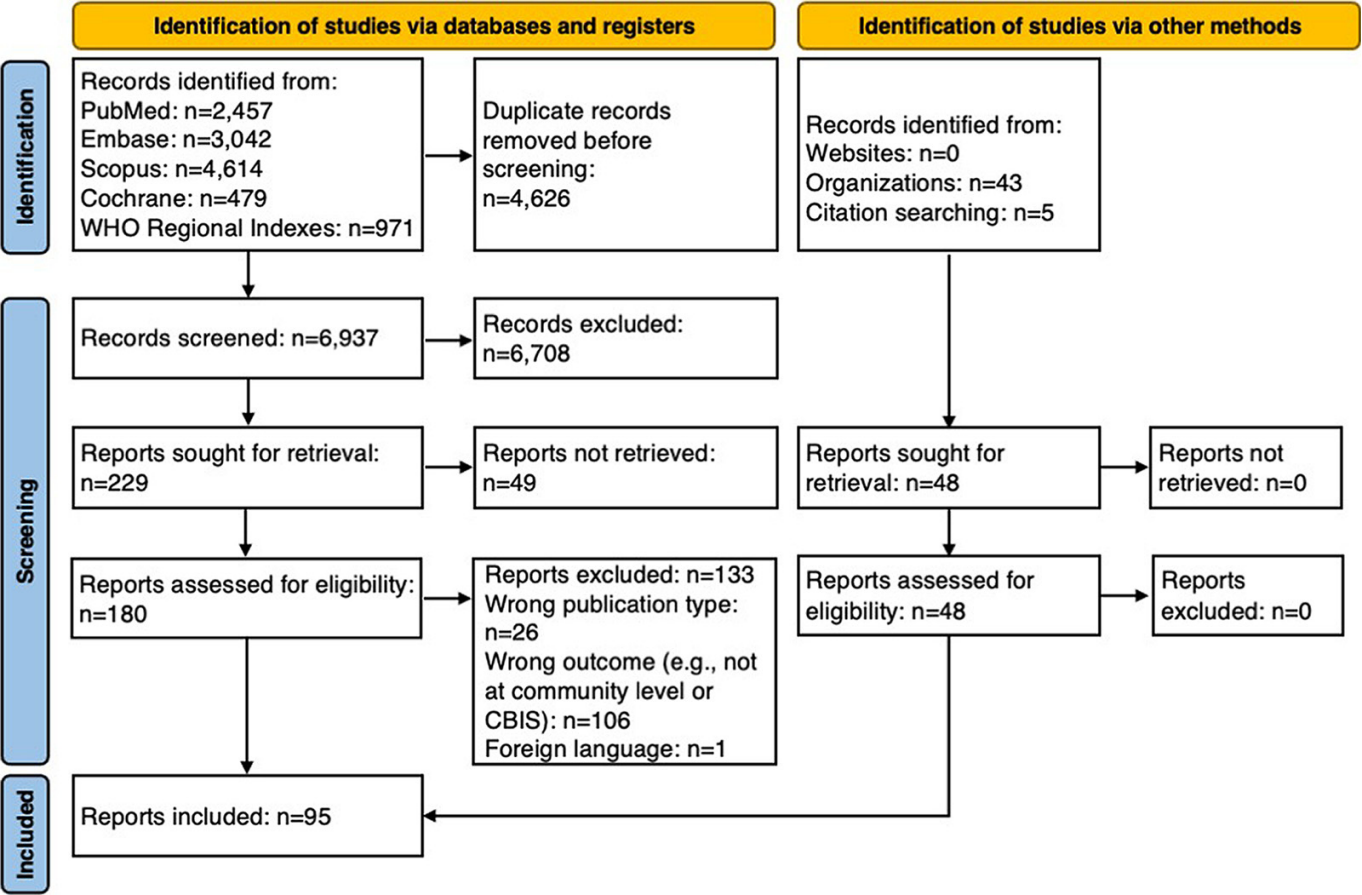
Scoping Review Search Results Using the PRISMA 2020 Flow Diagram

Figure 1 presents the search results. For the peer-reviewed literature, 6,937 unique articles were identified after deduplication. After title and abstract review, 229 articles remained. The full-text review resulted in 133 articles being excluded. Reasons for exclusion included inability to access the full-text article, article was not in English, the article was not about or there was no discussion around a CBIS, and/or the CBIS was not health related. From the peer-reviewed literature search, 47 original research articles met the inclusion criteria and were included in the review. Five additional peer-reviewed original research articles were identified by study investigators through citation searching, which resulted in the inclusion of 52 peer-reviewed articles. For the gray literature, 43 met the inclusion criteria and were included in the review. Seven of the peer-reviewed and gray literature reported on multiple countries. Overall, a total of 95 original research articles and gray literature were included in this scoping review.

Several articles reported on CBIS implementations across multiple countries or reported on the same CBIS country implementation. Therefore, the results are presented based on the CBIS implementation. In total, there are 105 CBIS implementations reported across the 95 included articles (Supplement 2).

### Community-Based Health Information Systems Implementations Characteristics and Context

**FIGURE 2:**
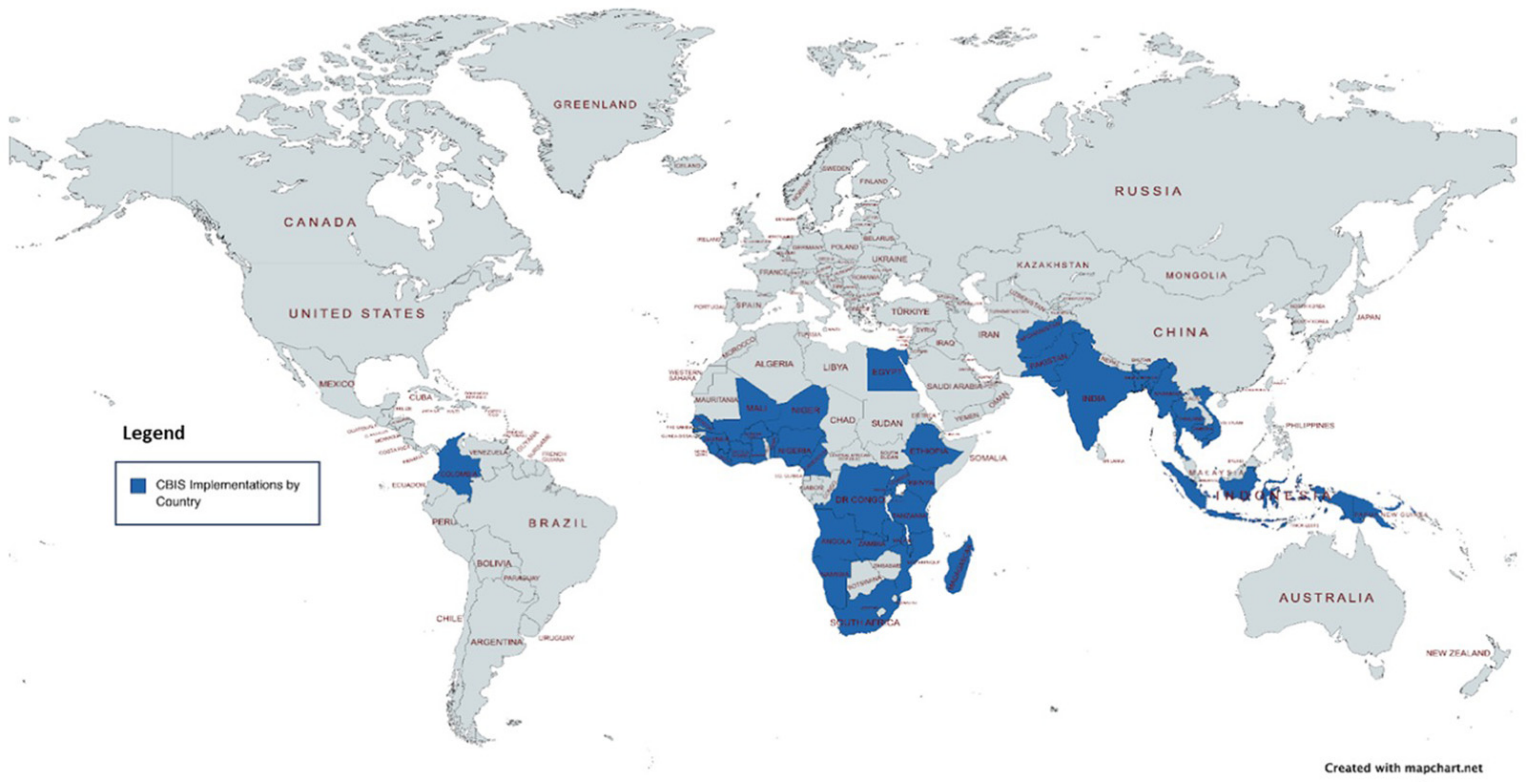
Geographic Distribution of Community-Based Health Information System Implementations From Scoping Review in 38 Countries

CBIS implementations (N=105) were spread across 38 countries (Figure 2). The majority of studies reported on CBIS implementations (n=78) in Africa, including Ethiopia,^16^^–^^23^ Malawi,^24^^–^^32^ Kenya,^31^^,^^33^^–^^36^ Uganda,^37^^–^^42^ South Africa,^8^^,^^43^^–^^46^ Mali,^47^^–^^50^ among others.^6^^,^^7^^,^^39^^,^^51^^–^^80^ Twenty-six CBIS implementations were reported in Asia, including India,^81^^–^^86^ Bangladesh,^10^^,^^87^^–^^89^ Papua New Guinea,^9^^,^^10^^,^^87^^,^^90^ Cambodia,^91^^–^^93^ Myanmar,^87^^,^^94^^,^^95^ among others.^96^^–^^100^^,^^103^ Only 1 CBIS implementation was reported in South America: Colombia.^87^

The majority of CBIS implementations (55.2%, n=58) reported using both paper and digital components for data collection and reporting (Table). Few (16.2%, n=17) were paper only, which means that both data collection and reporting were done using paper registries and reports.^16^^–^^19^^,^^22^^–^^25^^,^^29^^,^^32^^,^^37^^,^^43^^,^^46^^,^^52^^,^^60^^,^^91^^,^^99^ Less than one-third (28.6%, n=30) of CBISs were fully digitized, meaning that data collection and reporting were both digitized. ^7^^,^^10^^,^^21^^,^^26^^,^^28^^,^^38^^,^^40^^,^^41^^,^^44^^,^^45^^,^^55^^,^^56^^,^^62^^,^^64^^,^^68^^–^^70^^,^^81^^,^^82^^,^^84^^,^^85^^,^^87^^,^^89^^,^^94^^,^^95^^,^^97^^,^^98^^,^^101^ About one-third (30.5%, n=32) of CBISs monitored and tracked PHC-related services,^5^^–^^7^^,^^18^^,^^19^^,^^21^^,^^23^^,^^24^^,^^29^^,^^31^^,^^34^^,^^38^^–^^40^^,^^44^^,^^46^^,^^47^^,^^50^^,^^58^^,^^68^^,^^70^^,^^71^^,^^83^^,^^90^^,^^91^^,^^99^^,^^102^ 25.7% (n=27) focused on MNCH related services,^16^^,^^17^^,^^22^^,^^25^^,^^26^^,^^28^^,^^32^^,^^35^^,^^37^^,^^40^^,^^41^^,^^43^^,^^49^^,^^52^^,^^54^^,^^60^^,^^64^^,^^65^^,^^69^^,^^84^^–^^86^^,^^88^^,^^96^^,^^97^^,^^101^ and 2.9% (n=3) were nutrition-focused, looking at cases of malnutrition and food security, which were all in conjunction with MNCH.^17^^,^^27^^,^^97^ For infectious diseases, around 27.6% of CBISs focused on malaria,^20^^,^^30^^,^^33^^,^^42^^,^^48^^,^^51^^,^^53^^,^^61^^,^^63^^,^^66^^,^^67^^,^^72^^–^^81^^,^^92^^–^^95^^,^^100^^,^^101^^,^^103^^–^^105^ 10.5% tracked vital events (i.e., CRVS),^9^^,^^10^^,^^27^^,^^45^^,^^87^^,^^89^^,^^98^ 4.8% focused on HIV/AIDS,^36^^,^^40^^,^^55^^,^^65^^,^^101^ and 1.9% focused on Ebola.^56^^,^^57^

The majority of CBISs were implemented by donors/nongovernmental organizations and governments (77.1%, n=81); this largely meant that governments may have been supporting implementation of the CBIS, but external funding was provided to finance it. Less than 5% (4.8%, n=5) of the CBISs were solely government owned,^16^^,^^34^^,^^46^^,^^67^^,^^89^ and 17.2% (n=18) were led by donors/nongovernmental organizations.^17^^,^^31^^,^^38^^,^^45^^,^^49^^,^^56^^,^^62^^,^^64^^,^^75^^,^^83^^,^^84^^,^^86^^–^^88^^,^^91^^,^^94^^,^^95^^,^^99^ Most of the CBISs were being implemented at the subnational level (61.9%, n=65). Less than a quarter (23.8%, n=25) described CBISs that were integrated at the national level, which were largely vertical CBISs that had dedicated funding mechanisms (i.e., for malaria and HIV).^30^^,^^33^^,^^39^^,^^42^^,^^48^^,^^51^^,^^53^^,^^55^^,^^61^^,^^67^^,^^69^^,^^72^^,^^73^^,^^75^^,^^79^^,^^80^^,^^93^^,^^100^^,^^102^^,^^103^^,^^105^ All of the CBIS implementations that were integrated into the national level included some level of digitization.

### Community-Level Data Collection and Reporting Processes

Community-level data were largely collected by CHWs across the included articles; other community leaders, such as the village chief,^62^ community mobilizers,^56^ religious/faith leaders,^56^^,^^87^ traditional birth attendants,^87^ and nurses or midwives, were also identified as collectors of community data.^99^

**FIGURE 3:**
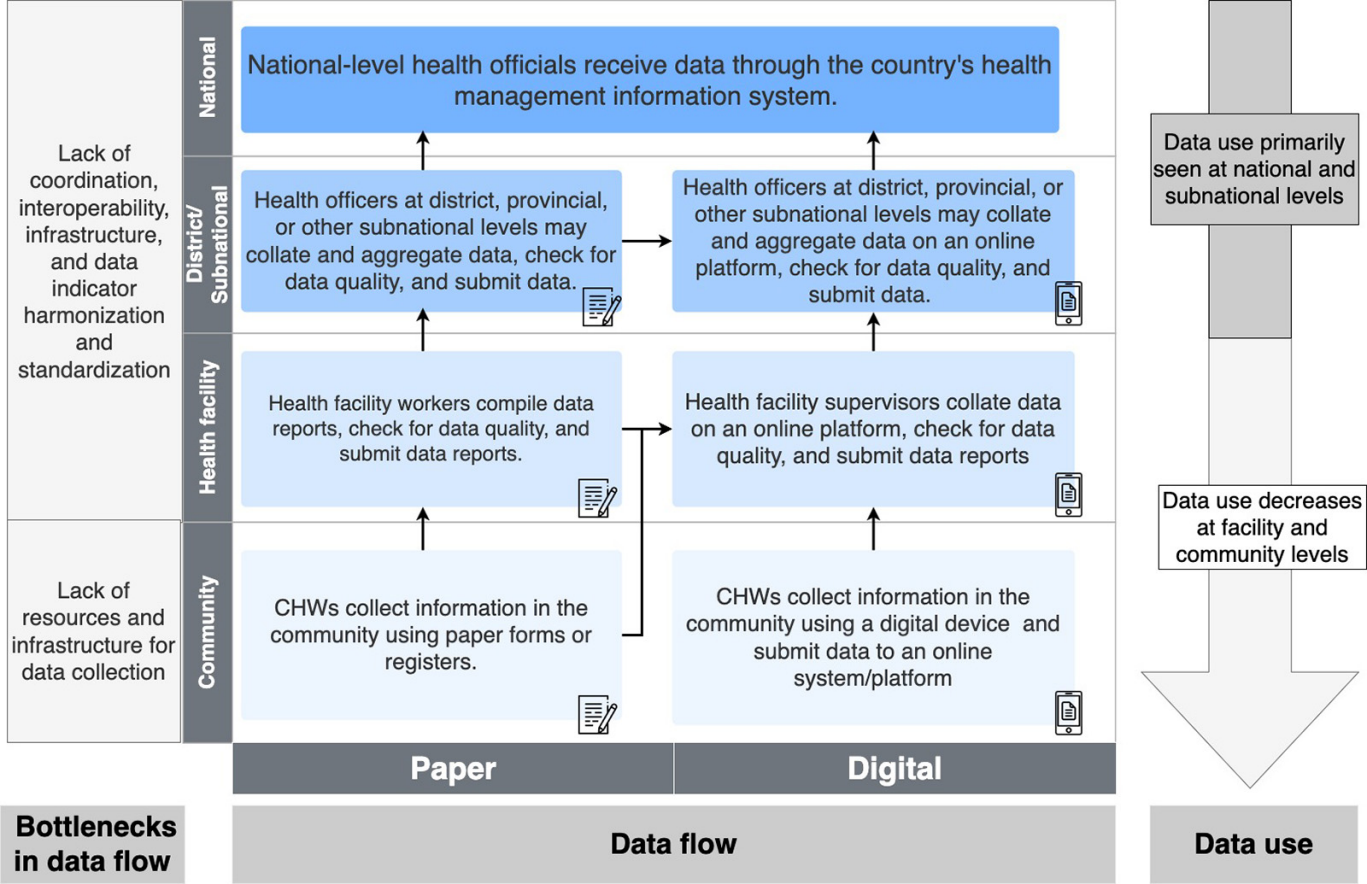
Data Flow Process in a Community-Based Health Information System^a^ ^a^ Illustrates multiple points where paper systems can integrate with digital ones, highlights the bottlenecks in data flow at each level of the health system, and demonstrates the decreased use of data at lower tiers of the health system, as identified through research findings.

Data reporting from the community level to the health system varied. The overall data flow process within a CBIS is summarized in Figure 3, which depicts that data were typically collected in the community at the household level, primarily by CHWs. These data were reviewed, aggregated, and submitted to PHC facilities, where they could be further aggregated and submitted to district-level and other subnational-level administrative units before reaching national-level integration. ^20^^,^^24^^,^^25^^,^^31^^,^^32^^,^^39^^,^^43^^,^^49^^,^^51^^,^^53^^,^^54^^,^^58^^,^^61^^,^^63^^,^^65^^,^^67^^,^^73^^–^^77^^,^^79^^,^^83^^,^^87^^–^^92^^,^^96^^,^^99^^,^^100^^,^^104^ With digitization, data reporting could be further streamlined where data could be immediately submitted and accessed at different levels of the health system from the point of community-level data collection. For example, the Maternal and Child Survival Program, whose implementation was described in 4 African countries, highlighted that data submitted using paper-based forms would often undergo data aggregation at both the health facility and district level before the data were transmitted to the national level. In comparison, digital collection of data can expedite this process as aggregate-level data reports can be automatically generated and accessed at all relevant levels of the health system for decision-making.^39^ However, the data reporting process outlined in Figure 3, which shows the ideal scenario of data reaching national-level integration, was not always seen in practice. CBISs that were focused on vertical programming, such as malaria or HIV, were more likely to report being integrated into a national HIS.^30^^,^^48^^,^^55^

Data reporting from the community level to the health system varied.

For paper-based CBISs, the Real-Time Mortality Monitoring Project in Malawi demonstrated how CHWs would collect household-level data and report the data to their supervisor, who would then review and share the data with the district-level coordinator; these data would then be photocopied to share with the National Statistics Office who had dedicated staff to collect and review the CHW data.^25^ However, this was not often the case seen for paper-based CBISs, where paper-based reporting could be hampered by a range of challenges, including not having access to the very registers and reporting forms to submit data to health facilities, registers and reporting forms being vulnerable to damage and loss due to weather, and delays in submitting data in a timely manner when data were available. As such, these challenges could have impeded the ability of a paper-based CBIS being able to reach national-level integration.^16^^,^^23^^,^^86^^,^^91^

For mixed or digital CBISs, data could automatically be uploaded from digital devices to a centralized platform or server where data were further consolidated, reviewed, and analyzed.^6–8^^,^ ^10^^,^^21^^,^^38^^,^^47^^,^^48^^,^^52^^,^^55^^,^^62^^,^^68^^,^^70^^,^^81^^,^^82^^,^^86^^,^^87^^,^^94^^,^^101^^,^^106^^,^^107^ Among cases where the CBIS was partially digitized, monitoring and evaluation officers or data clerks were reported to assist with manual data entry at the health facility levels.^27^^,^^35^^,^^44^^,^^65^^,^^78^^,^^102^ In a cluster-randomized controlled trial in Zambia, mobile-collected data to support integrated community case management among CHWs who received the m-Health intervention were submitted to the DHIS-2 server where CHW supervisors were able to access data in real time and provide feedback to CHWs directly.^6^ In India, CHWs directly captured maternal and child health data on the ImTeCHO mobile web application, a fully digital system that was used to generate CHW performance reports, facilitate timely payments for CHWs based on performance, and for logistics management.^85^ In Ethiopia, while malaria-specific data were collected both manually and digitally at the community level, the national electronic Community Health Information System was being scaled to link collated data to a central network that connected health facilities and other administrative health units, enabling easy access to data at all levels of the health system.^20^

### Digitization-Related Challenges in Community-Level Data Collection and Reporting

Although digitized CBISs offer promise, challenges with electricity and connectivity infrastructure, insufficient training and digital literacy among the health workforce, and transitioning from paper-based to digital systems all impacted data collection, data quality, data flow, and reporting negatively.^6^^,^^44^^,^^74^^,^^88^ Further, the digital systems themselves may have caused further challenges. There was often a lack of interoperability between different systems that could have impacted the effective integration of community-level data.^44^^,^^74^^,^^88^^,^^100^ Additionally, given disparate systems, indicators that were being collected at the community level may not have been the same as what was reported in national or subnational-level HIS. For example, in Cameroon, it was reported that only 20% of the data collected by CHWs were captured in the national-level HIS largely due to differences in the reporting forms between what was collected at the community level and what was listed in the national HIS reporting form.^74^

### Drivers of Data Quality in Community-Based Health Information Systems

The literature identified several challenges around data collection, data completeness and availability, and data reporting that impacted data quality, which has also been widely documented in other literature around the use of HIS broadly.^108^^–^^110^ Data availability was constrained in some cases due to shortages and unavailability of paper-based and digital data collection tools, as well as the lack of training provided on using these tools.^20^^,^^31^^,^^33^^,^^37^^,^^58^^,^^65^ A review of CBIS data quality in Kenya and Malawi cited the lack of registers as a barrier to data reporting and data quality, noting that, in some cases, CHWs either purchased registers themselves or identified alternate ways to record community-level data (i.e., children’s school exercise books, cardboard boxes).^31^ Data entry errors at the time of data collection coupled with insufficient data review or validation processes also impacted data quality and underscored the need for data quality assessments/checks.^39^^,^^65^^,^^74^^,^^81^^,^^86^^,^^90^ In Uganda, it was reported that there was often insufficient time or resources to do data quality checks on the paper-based community-level data that were being collected by CHWs.^39^ In Malawi, authors emphasized a need for protocols to address data collection and data entry errors to strengthen data quality and the CBIS itself to improve provision of health services and programming.^32^

Data entry errors at the time of data collection coupled with insufficient data review or validation processes impacted data quality and underscored the need for data quality assessments/checks.

The paper-to-digital transition for CBISs also resulted in both improvements in and challenges with data quality. Although digitization could improve data quality given automation of data verification within the tools itself,^21^^,^^68^^,^^86^^,^^92^ the transition from paper to digital often resulted in the double burden of data reporting, wherein CHWs may have been recording data on paper and then manually transferring and digitizing it, which increased the workload of CHWs and could reduce data quality.^35^^,^^39^^,^^88^ In Bangladesh, it was reported that CHWs manually entered data from four different registers into DHIS-2, which saw challenges around data duplication, overreporting, and underreporting, as well as the increased workload burden placed upon CHWs.^88^ In contrast, a digital CBIS for TB and MNCH services piloted in Southern Ethiopia reported that integrating the digital tool within local and collaborative processes facilitated data quality given automated data validation and data quality checks, especially in contrast to the existing paper-based system.^21^

Fragmentation of the CBIS landscape also impacted data quality; there were multiple parallel and siloed reporting systems across different organizations and Ministries, with a lack of harmonization and coordination between them.^23^^,^^24^^,^^30^^,^^100^ This could have led to CHWs having to manage different registers, tools, and forms to collect data and submit reports. In Tanzania, CHWs noted that there was repetition across the forms they completed and recommended that the number of forms be consolidated to reduce duplication of data and impact on CHW workload.^59^ Additionally, health data indicators between systems may have been similar but not the same, making it difficult to validate and connect data.^23^^,^^24^^,^^35^^,^^37^^,^^100^ In Uganda, indicators on the community-level CHW registers did not match the health management information system indicators, emphasizing the need for harmonization of these indicators to better facilitate monitoring CHW performance, health services, and health outcomes.^37^ In Ethiopia, authors cited that the lack of harmonization and standardization of indicators negatively impacted data quality of the paper-based “Family Folders” CBIS, recommending that there be a “core set of indicators” to support indicator harmonization.^24^

The studies in this review also identified a number of strategies that could help improve data quality, primarily around the implementation of robust and largely digitized monitoring and evaluation systems that could identify strengths and gaps in CBIS data flow and facilitate real-time feedback on health system performance^32^^,^^35^^,^^59^^,^^65^^,^^70^^,^^96^; components of this also included consistently implemented data review meetings with CHWs and CHW supervisors to promote accountability.^58^^,^^63^^,^^71^^,^^91^^,^^105^

### Data Use

Limited availability and lack of trust in the quality of community-level data impeded ability to effectively use CBIS data for decision-making and toward improving community-level health services and health outcomes. As such, data use for decision-making was fairly limited, primarily occurring at the subnational and national levels (Figure 3). One significant challenge with leveraging community-level data at the national level was the fact that community-level data were often not integrated into the national HIS. For example, Ashwell et al. noted that CHWs submitted their reports to health facilities in Papua New Guinea, but health facilities in some provinces were not consistently including the data within their reports that they sent to the next level. This was reported to be because of data use; if community-level data were being used to monitor health services, trends, and CHW performance, then the data would be included.^90^ Further, it was reported in Papua New Guinea that when community-level data were included, the data did not allow for disaggregation or differentiation between community data versus health facility data, which impacted the ability to understand the impact of community-level health services.^90^

Limited availability and lack of trust in the quality of community-level data impeded ability to effectively use CBIS data for decision-making and toward improving community-level health services and health outcomes.

Data use at the community level was primarily focused on data review meetings with CHWs around data quality. In a few cases, CHWs were actively involved in the use of data to improve health services. For example, in Ethiopia, a study reported that engaging CHWs in analyzing data to measure progress, identify gaps, and develop solutions to community health problems demonstrated improvements in MNCH in their communities.^17^ In Malawi, CHWs were responsible for describing health information in each village every month, plotting performance graphs for each health indicator per village, and submitting aggregate data to the health center.^29^ Despite the clear benefits of data use at the community level, in practice, data use and decision-making by CHWs were largely informal and nonsystematic.^39^ Byrne et al. emphasized the importance of encouraging CHWs to regularly share their experiences in data collection and discuss data with each other to continually improve their engagement in CBIS implementation.^43^

CBIS data were also used largely by health facilities to review CHW performance and performance targets, resource allocation, and progress monitoring, as well as to improve service delivery practices.^102^ At the district level, data were largely used to improve program implementation and resource allocation. For example, district health management teams in Burkina Faso used data to support health worker coaching and performance improvement, disease surveillance, technical repairs, and trainings.^70^ Dashboard data from DHIS-2 were used by district-level staff to examine health trends and develop MNCH strategies in the Democratic Republic of Congo.^39^ In Uganda, monthly district reports on malaria indicators were generated to provide feedback to health facilities on data quality and stock commodity allocation.^42^

At subnational and national levels, data use was primarily seen among vertical CBISs. CRVS-specific CBIS data were used to understand population status and prompt the creation of birth and death certificates.^87^^,^^89^ In Nigeria, community-level data from a malaria CBIS were ultimately submitted to the national-level DHIS-2, where data were analyzed at routine monitoring and evaluation meetings and strategic health decisions around malaria control interventions were made.^53^ Data use was also seen through the use of dashboards for routine surveillance, stock management, or CHW performance tracking to help subnational and national level officials set health and performance targets, mobilize resources, and ensure the appropriate CHW support was provided.^63^^,^^70^^,^^80^^,^^81^^,^^88^^,^^100^

Digitization was reported to improve access to and availability of data for data use at all levels of the health system, as data can become available near real time for use to facilitate consistency, completeness, and timeliness of the data collected.^7^^,^^19^^,^^21^^,^^26^^,^^41^^,^^46^^,^^53^^,^^86^^,^^101^ However, despite improved data availability, the ability to connect and integrate community-level data with national or subnational-level HIS for decision-making was still limited.^38^^,^^42^^,^^48^^,^^105^ The literature emphasized the need for interoperability, improved data quality, increased digital literacy training, improved network and connectivity infrastructure, and human resources to enable effective data use. ^26^^,^^41^^,^^47^^,^^97^

## DISCUSSION

With the renewed agenda around the importance of investing in community health systems and augmented by the fact that around half of the global population does not have access to essential health services, it is critical to consider the role that CBISs play in advancing PHC and universal health coverage.^111^^,^^112^ This scoping review identified 95 articles/documents reporting on 105 paper, digital, and mixed CBIS implementations globally. The majority of CBISs included both paper-based and digital components, with the initial community-level data collection often being paper-based and the data becoming further digitized as it flowed through the levels of the health system. Data flow was largely unidirectional, going from the largely CHW-collected household data to being further aggregated and reported to the health facilities/subnational levels. In some cases of CBIS implementations, there was national-level integration, but this was context dependent and largely seen among vertical disease-specific CBISs. Data use was primarily seen at the subnational and national levels, with limited use being reported at the community level.

It is critical to consider the role that CBISs play in advancing PHC and universal health coverage.

Challenges around the use of CBISs largely mirrored what is seen in the literature with the use of routine HIS. Inconsistent data quality, lack of trust in the data being reported, lack of interoperability between HIS, and inadequate standardization or harmonization of health indicators were all reported as key challenges with the use of CBISs. However, CBISs face the unique challenge that community-level data are ultimately not being consistently integrated into HIS at the subnational or national levels. Health facilities may collect community-level data but may not report it further within the health system, or they may combine community-level data with health facility data, thereby limiting or removing understanding of the community-level context (as it is indiscernible from health facility data).^74^^,^^90^ As such, the lack of consideration and integration of community-level data impedes the utility of CBIS for decision-making and renders it to primarily function as a way in which data are collected and reported but not necessarily used.

Community-level data through CBISs are essential for understanding what the health needs of the population are and can directly support the achievement of universal health coverage. Investing in CBISs and community health is needed to build resilient health systems, especially in light of the recent pandemic and epidemics. During the COVID-19 pandemic and Ebola epidemic, CHWs and community health systems played a critical role in contact tracing, case management, and provision of health services, and these services and data were critical to understanding what the health contexts and needs were.^113^^–^^115^ However, despite their role and importance, there is insufficient and inadequate coordination, investment, and prioritization of community health systems within PHC.

Digitization is often seen as an opportune solution, given the potential it has in facilitating access to quality data collected in real time. The transition of CBISs from being paper based to including digital components or becoming fully digitized has yielded some improvements in timeliness of data reporting, data quality, potential for integration into subnational and national HISs, and potential increased data use for decision-making. However, the potential of digitization will not overcome persistent infrastructural and systemic challenges exacerbated by the lack of sufficient investment in community health systems. Challenges around human resources and CHW workforce retention, the enabling digital environment of mobile/network connectivity and electricity, the need for training—not just around digital literacy but also on data collection, reporting, and use—provision of supportive supervision to CHWs, among others, all impact the ability to collect and report quality community-level data.

### Strengthening Community Health Systems Will Strengthen Community-Based Information Systems

Without a strengthened community health system, the role and value of CBISs will remain limited in scope. Currently, the community health landscape is rife with fragmentation. Community health programs themselves depend on donor funding largely linked to siloed disease/health-specific vertical programming.^116^ This directly and inevitably impacts the CBIS landscape, where we concurrently are seeing fragmentation as well, with disparate paper-based, digital, or mixed CBISs that are driven by disease/health-specific vertical programming. This results in CHWs having to manage multiple, sometimes duplicative, CBISs for different disease areas, further exacerbating the workload burden of an already overburdened and largely unpaid workforce.^4^^,^^117^ These CBISs may overlap in scope, where they may be collecting similar but not the same data indicators, so data between CBISs cannot necessarily be fully linked or directly compared.^118^ Further, these CBISs are often not interoperable with other CBISs, let alone with a subnational or national HIS. There are many pilot-level studies and evaluations that reported on data collection at the community level, many of which were included in this review and provided a strong proof-of-concept, but they too were not necessarily always developed or implemented with active government engagement or with consideration of further integration of the community-level data within the broader context of the local health system. Broadly, community health systems and donor investments need to ensure the integration of PHC services to foster alignment and country-led coordination and reduce further fragmentation of community health systems, including CBISs.^116^^,^^119^

There have been efforts made and calls to action to further advocate for CBISs. The 2019 World Health Assembly resolution WHA72.3 endorsed the need for alignment of data and digital efforts to optimize CHW programs and develop a stronger evidence base for CHW impact.^120^ In 2021, the Health Data Collaborative—a collaborative network that aligns technical and financial resources toward strengthening country HIS—released the report, “Guidance for Community Health Workers Strategic Information and Service Monitoring,” in response to WHA72.3 to provide a defined set of standardized indicators with the ultimate goal of supporting monitoring and evaluation of community health systems, reduction of fragmentation, reduction of reporting burden, and harmonization and integration of indicators into national/subnational HIS.^118^ Additionally, efforts have been led by the Community Health Impact Coalition—an advocacy-focused network focused on professionalizing and supporting CHWs—to harmonize and connect community-level data across 8 countries represented within the Coalition.^121^ These are all critical steps toward improving CBISs with the goal of strengthening community health systems.

CBISs can play a needed role in identifying where investments in community health systems should be directed. Currently, community-level health data are not necessarily being integrated or used, but further investing in CBISs can directly provide the requisite data and information on the current health contexts to guide investments and what services/programming needs to be provided. Digitization of CBISs is seen as a possible solution with the ability to collect and leverage real-time data. However, the question arises on the effectiveness of digitization within the context of a fragmented community health system. Ultimately, the lack of investment in CBISs and their limited integration and use to facilitate improvements in health services and programming impede our ability to understand the functioning of community health systems and where investments may be needed. Additionally, there is insufficient evidence around the impact of CBISs, as the role community-level data play is largely underemphasized and underreported in the broader literature, calling for the need for further studies on how CBISs can be better integrated within the larger health system for increased efficiency and use.

CBISs can play a needed role in identifying where investments in community health systems should be directed.

### Strengths and Limitations

This study employed a comprehensive search strategy, including both peer-reviewed and gray literature, to document and understand the range of CBIS implementations. However, the amount of information and evidence regarding the CBIS varied between articles. The study also primarily relied on open-access and English-only articles, which precluded potentially relevant articles from inclusion due to issues of accessibility.

## CONCLUSION

There is limited evidence on the effectiveness of CBIS in LMICs within the literature. This scoping review described the implementations and documented how community-level data has been integrated and used to support community health outcomes. Despite the increase in digitization of CBISs, it is important to focus on the broader ecosystem to strengthen data collection and reporting systems and address challenges around training, connectivity, and integration of health services to improve the quality and use of community-level data.

**TABLE.** Descriptive Characteristics of Community-Based Information System Implementations

	**No. (%)** **(N=105)**
Type	
Paper only	17 (16.2)
Digital only	30 (28.6)
Mixed (paper and digital components)	58 (55.2)
Health domain	
Primary health care	32 (30.5)
Maternal, newborn, and child health	27 (25.7)
Infectious disease (e.g., malaria, HIV/AIDS, Ebola)	36 (34.3)
Nutrition	3 (2.9)
Civil Registration and Vital Statistics	11 (10.5)
Other	2 (1.9)
Scale	
National	25 (23.8)
Subnational	65 (61.9)
Pilot	15 (14.3)
Type of implementor (funder)	
Government	5 (4.8)
Donor/nongovernmental organization	18 (17.2)
Mixed	81 (77.1)
Unclear	1 (1.0)
Program approach	
Vertical	77 (73.3)
Horizontal	28 (26.7

## Supplementary Material

GHSP-D-23-00429-supplements.pdf

## Notes

**Peer Reviewed**

**First Published Online:** April 17, 2024.
