# Supplementary material for: How Can Community Data Be Leveraged to Advance Primary Health Care? A Scoping Review of Community-Based Health Information Systems
Source: Glob Health Sci Pract. 2024 Apr 29;12(2):e2300429. doi: 10.9745/GHSP-D-23-00429 (PMC11057800; doi:10.9745/GHSP-D-23-00429)
Supplement: GHSP-D-23-00429-supplements.pdf [file GHSP-D-23-00429-supplements.pdf]

Supplement to: Pandya S, Kan L, Parr E, Twose C, Labrique AB, Agarwal S. How can community-level data be leveraged to advance primary health care? A scoping review of community-based health information systems. *Glob Health Sci Pract*. 2024;12(2):e2300429. https://doi.org/10.9745/GHSP-D-23-00429

Supplement 1. Scoping Review Search Strategy

Databases Utilized for Scoping Review Search Strategy

Five databases were utilized to perform the search strategy on November 15, 2021:

- PubMed
- Embase
- Scopus
- Cochrane
- WHO Regional Indexes

Search Strategy.

The search strategy combined searches for three primary concepts:

(1) community-based health information systems
  a. Terms included: *community based health information system, community health monitor, health information system, routine health information system, community health management, performance monitoring, reporting system, community surveillance, health information exchange, community health workers, registers, civil registration and vital statistics*

(2) low and middle income countries
  a. Terms included country names as well as: *low and middle income countries, lami countries, less developed countries, less developed economies, deprived countries, lesser developed countries, low gdp, low gnp, low gross domestic, low income countries, middle income countries, lower income countries, poorer countries, under developed countries, under served countries.*

Supplement 2. List of Included Articles, Country of CBIS Implementation, and Type of CBIS Implementation (n=105 CBIS implementations).

Author & Year	Country	Type of CBIS
Zaidi et al (2020) [97]	Afghanistan	Digital
Angola, PMI (2021) [73]	Angola	Mixed
Adair et al (2020) [87]	Bangladesh	Mixed
Begum et al (2019) [88]	Bangladesh	Mixed
Hazard et al (2020) [10]	Bangladesh	Mixed
Uddin et al (2019) [89]	Bangladesh	Digital
Benin, PMI (2021) [67]	Benin	Mixed
Benin, USAID (2021) [68]	Benin	Digital
Gilmartin et al (2015) [69]	Burkina Faso	Digital
Burkina Faso, Exemplars in Global Health (2021) [70]	Burkina Faso	Digital
Burma, PMI (2021) [103]	Myanmar (Burma)	Mixed
Oum et al (2005) [91]	Cambodia	Paper
Cambodia, PMI (2021) [92]	Cambodia	Mixed
Mellor et al (2013) [93]	Cambodia	Mixed
Cameroon, PMI (2021) [74]	Cameroon	Mixed
Adair et al (2020) [87]	Colombia	Mixed
Côte d'Ivoire, PMI (2021) [75]	Côte d'Ivoire	Digital
Democratic Republic of Congo (DRC), PMI (2021) [61]	DRC	Mixed
Rubenstein et al (2015) [62]	DRC	Digital
USAID & MCS Program (2019) [39]	DRC	Mixed
USAID & MCS Program (2019) [39]	Egypt	Mixed
Ethiopia, USAID (2019) [102]	Ethiopia	Mixed
Hirvonen et al (2020) [16]	Ethiopia	Paper
Karim et al (2018) [17]	Ethiopia	Paper
Ethiopia, MEASURE Evaluation (2012) [18]	Ethiopia	Paper
University of Washington Global Health Start Program (2014) [24]	Ethiopia	Paper
Ejeta et al (2020) [19]	Ethiopia	Paper
Ethiopia, PMI (2021) [20]	Ethiopia	Mixed
Mengesha et al (2018) [21]	Ethiopia	Digital
Yaya et al (2015) [22]	Ethiopia	Paper
Damtew et al (2013) [23]	Ethiopia	Paper
Ghana, PMI (2021) [63]	Ghana	Mixed
Willcox et al (2019) [64]	Ghana	Digital

Supplement 2. List of Included Articles, Country of CBIS Implementation, and Type of CBIS Implementation (n=105 CBIS implementations).

Author & Year	Country	Type of CBIS
Mutale (2013) [58]	Ghana	Paper
Guinea, PMI (2021) [51]	Guinea	Mixed
Rajvanshi et al (2021) [81]	India	Digital
Peiris et al (2019) [82]	India	Digital
Quraishy (2006) [83]	India	Digital
Thomas et al (2012) [84]	India	Digital
Modi et al (2019) [85]	India	Digital
Nguyen et al (2015) [86]	India	Digital
Rambu Ngana et al (2012) [99]	Indonesia	Paper
Kenya, PMI (2021) [33]	Kenya	Mixed
Regeru et al (2020) [31]	Kenya	Mixed
Jeremie et al (2014) [34]	Kenya	Digital
Kenya, MEASURE Evaluation (2014) [35]	Kenya	Paper
Mushamiri et al (2015) [36]	Kenya	Digital
Liberia, PMI (2021) [76]	Liberia	Mixed
Madagascar, PMI (2021) [77]	Madagascar	Mixed
Amouzou et al (2014) [25]	Malawi	Paper
Blaschke et al (2009) [26]	Malawi	Digital
Konopka et al (2015) [27]	Malawi	Mixed
Malawi, SC4CCM (2013) [28]	Malawi	Digital
Kubalalika (2018) [29]	Malawi	Paper
Malawi, PMI (2021) [30]	Malawi	Mixed
Regeru et al (2020) [30]	Malawi	Mixed
Yourkavitch et al (2016) [32]	Malawi	Paper
Kirk et al (2021) [47]	Mali	Mixed
Mali, PMI (2021) [48]	Mali	Mixed
Stewart et al (2001) [49]	Mali	Digital
Whidden et al (2018) [50]	Mali	Mixed
PMI (2018) [104]	Mozambique	Mixed
Malaria Consortium (2022) [101]	Mozambique	Digital
Mozambique, PMI (2021) [103]	Mozambique	Mixed
Adair et al (2020) [87]	Myanmar (Burma)	Mixed
*Oo et al (2021a) *** [94]	Myanmar (Burma)	Digital
*Oo et al (2021b) *** [95]	Myanmar (Burma)	Digital
USAID & MCS Program (2019) [39]	Namibia	Mixed
Niger PMI (2021) [79]*8*	Niger	Mixed
Asangansi et al (2013) [7]	Nigeria	Digital
Nigeria, PMI (2021) [67]	Nigeria	Mixed
Nyangara et al (2018) [52]	Nigeria	Paper
Durrani et al (2019) [98]	Pakistan	Digital
Zaidi et al (2020) [97]	Pakistan	Digital

Supplement 2. List of Included Articles, Country of CBIS Implementation, and Type of CBIS Implementation (n=105 CBIS implementations).

Author & Year	Country	Type of CBIS
Adair et al (2020) [87]	Papau New Guinea	Mixed
Firth et al (2021) [9]	Papau New Guinea	Mixed
Hazard et al (2020) [10]	Papau New Guinea	Mixed
Ashwell et al (2010) [90]	Papau New Guinea	Mixed
Rwanda, PMI (2021) [79]	Rwanda	Mixed
Muhoza et al (2021) [71]	Senegal	Mixed
Senegal, PMI (2021) [72]	Senegal	Mixed
O'Connor et al (2019) [54]	Sierra Leone	Mixed
Braa et al (2010) [55]	Sierra Leone	Digital
Jalloh et al (2020) [56]	Sierra Leone	Digital
Thomas et al (2019) [57]	Sierra Leone	Mixed
Byrne et al (2007) [43]	South Africa	Paper
Odendaal et al (2020) [44]	South Africa	Digital
Walker et al (2018) [40]	South Africa	Digital
Igumbor et al (2020) [56]	South Africa	Digital
Mash et al (2020) [46]	South Africa	Mixed
Mutale (2013) [58]	Tanzania	Paper
Tanzania, PMI (2021) [80]	Tanzania	Mixed
Ramsey et al (2013) [60]	Tanzania	Paper
Tanzania, MEASURE Evaluation (2014) [59]	Tanzania	Mixed
Thailand, PMI (2021) [100]	Thailand	Mixed
Nanyonjo et al (2020) [37]	Uganda	Mixed
Uganda, Exemplars in Global Health (2021) [38]	Uganda	Digital
USAID & MCS Program (2019) [39]	Uganda	Mixed
Walker et al (2018) [40]	Uganda	Digital
Tumusiime et al (2014) [41]	Uganda	Digital
Uganda, PMI (2021) [42]	Uganda	Mixed
Tuan et al (2015) [96]	Vietnam	Mixed
Flora et al (2017) [5]	Kenya	Mixed
Hamainza et al (2014) [66]	Zambia	Mixed
Biemba et al (2017) [6]	Zambia	Mixed
Zambia, MEASURE Evaluation (2014) [65]	Zambia	Mixed

Notes:
** Paper-based CBIS refers to paper-based data collection and reporting; digital CBIS refers to one that has digitized data collection and reporting; a mixed CBIS is one that includes both paper-based and digital tools for data collection and reporting.*
*** These two articles reported on the same CBIS implementation.*
